# Diet and Exercise in the Treatment of Fatty Liver

**DOI:** 10.1155/2012/257671

**Published:** 2011-09-18

**Authors:** Faidon Magkos, Jean-Marc Lavoie, Konstantinos Kantartzis, Amalia Gastaldelli

**Affiliations:** ^1^Division of Endocrinology, Diabetes, and Metabolism, Beth Israel Deaconess Medical Center, Harvard Medical School, Boston, MA 02215, USA; ^2^Department of Kinesiology, University of Montreal, C.P. 6128, Succursale Centre Ville, Montreal, Canada QC, H3C 3J7; ^3^Division of Endocrinology, Diabetology, Nephrology, Vascular Disease and Clinical Chemistry, University of Tübingen, Department of Internal Medicine, Otfried-Müller-Straße 10, 72076 Tübingen, Germany; ^4^Research Director Stable Isotope Laboratory, Institute of Clinical Physiology, CNR, Via Moruzzi 1, 56100 Pisa, Italy

In recent years, we came to realize that obesity, broadly defined as increased body mass index or increased total body fat, is not necessarily associated with metabolic dysfunction and greater risk for cardiometabolic disease. In fact, there are several obese persons who are “metabolically healthy,” as there are nonobese persons who are “metabolically abnormal.” Although the reason(s) underlying this phenomenon are still not entirely clear, a number of studies conducted over the past several years indicate that the anatomical location of excess fat is more important than total body adiposity in determining metabolic outcomes. 

Ectopic fat accumulation, particularly in the liver, is frequently observed in obese persons and is strongly associated with metabolic dysfunction, including multiorgan insulin resistance and dyslipidemia. Intrahepatic fat, possibly more than visceral or intramyocellular fat, may thus be a prominent factor modifying the metabolic risk associated with increasing whole-body adiposity. However, cause-and-effect relationships have not yet been established, and it is also possible that intrahepatic triglyceride content is not a determinant but merely a marker of metabolic health. 

Understanding the regulation of fat accumulation in the liver will thus have important implications in both research and clinical practice. Little is known regarding the specific effects of lifestyle factors such as diet and exercise in regulating the accumulation of fat in the liver and its depletion thereof. In this special issue, we have invited a few papers in an attempt to partly fill this gap in our knowledge. 

In the first paper of this issue, “*Putative factors that may modulate the effect of exercise on liver fat: insights from animal studies*,” several studies in animals are reviewed in order to highlight putative factors that may modulate the effect of exercise on liver fat. This includes the fat content of the diet (exercise appears to be more effective under high-fat feeding), the role of concurrent exercise-induced loss of body weight or visceral fat, sex (males versus females), prandial status (fasted versus fed), and the duration of training, as well as the time elapsed from the last bout of exercise. The potential importance of these factors in modifying the exercise-induced changes in liver fat has not yet been formally tested in man, thereby providing a wide array of opportunities for future research. The second paper of this issue, “*Nafld, estrogens, and physical exercise: the animal model*,” focuses on the effects of exercise on liver fat in relation to estrogen availability. Estrogen deficiency, such as that occurring naturally after menopause in women, is strongly associated with fatty liver in animals. Exercise training exerts an estrogenic-like effect on the expression of genes involved in hepatic lipid metabolism and is a powerful means for preventing liver fat accumulation in estrogen-deficient animals. The third paper of this special issue, “*Dietary conjugated linoleic acid and hepatic steatosis: species specific effects on liver and adipose lipid metabolism and gene expression*,” reviews the effects of dietary conjugated linoleic acid on liver fat content and hepatic and adipose tissue fatty acid metabolism in animals. Conjugated linoleic acids, particularly the *trans*-10, *cis*-12, lead to hepatic steatosis owing to increased de novo lipogenesis and increased hepatic fatty acid uptake, at rates far exceeding the rates of disposal of intrahepatic fatty acids towards oxidation, esterification, and triglyceride export. 

The fourth paper of this issue, “*Effects of exercise training on molecular markers of lipogenesis and lipid partitioning in fructose-induced liver fat accumulation*,” examines the effects of exercise training on liver fat in starved and subsequently fructose-refed animals. Fructose, a simple sugar, is a potent dietary trigger for liver fat accretion. Exercise training in this model is not able to reverse the fructose-induced changes in lipogenic enzymes and does not reduce intrahepatic fat content. Thus, contrary to the large body of evidence demonstrating that exercise is effective in alleviating hepatic steatosis induced by high-fat feeding, exercise is not able to reverse the changes induced by fructose feeding. The final paper of this special issue, “*Exercise and omega-3 polyunsaturated fatty acid supplementation for the treatment of hepatic steatosis in hyperphagic OLETF rats*,” evaluates the effects of exercise on a hyperphagic model of obesity, with or without concurrent omega-3 polyunsaturated fatty acid supplementation. Exercise training in this animal model alleviates hepatic steatosis even under low-fat feeding conditions, predominantly by increasing hepatic fatty acid oxidation, whereas supplementation with omega-3 fatty acids slightly increases liver-fat content and attenuates the liver-fat-depleting effect of exercise. It is noteworthy that omega-3 fatty acid supplementation in this study accounted for only 3% of total dietary energy, whereas in several previous studies showing that omega-3 fatty acids reduce liver fat the supplement was administered at much greater doses. 

Research presented and reviewed in this special issue not only highlights the independent effects of exercise and diet on liver fat accumulation but also, more importantly, raises the intriguing possibility of interactive effects between exercise and diet on the mechanisms regulating liver fat accretion and depletion. It seems that several dietary factors are able to either augment or attenuate the intrahepatic triglyceride-depleting effect of exercise. 

*Faidon Magkos* *Faidon Magkos* *Jean-Marc Lavoie* *Jean-Marc Lavoie* *Konstantinos Kantartzis* *Konstantinos Kantartzis* *Amalia Gastaldelli* *Amalia Gastaldelli*

